# Case Report - Multinodular goiter in a patient with Congenital Hypothyroidism and Bannayan-Riley-Ruvalcaba syndrome: the possible synergic role of TPO and PTEN mutation

**DOI:** 10.3389/fendo.2023.1205785

**Published:** 2023-06-08

**Authors:** Gaia Vincenzi, Ilenia Teresa Petralia, Marco Abbate, Giulia Tarantola, Silvia Laura Carla Meroni, Riccardo Maggiore, Gilberto Mari, Maria Grazia Patricelli, Marco Schiavo Lena, Graziano Barera, Maria Cristina Vigone

**Affiliations:** ^1^ Department of Pediatrics, Endocrine Unit, IRCCS San Raffaele Scientific Institute, Milan, Italy; ^2^ Department of Pediatrics, Endocrine Unit, Vita-Salute San Raffaele University, IRCCS San Raffaele Scientific Institute, Milan, Italy; ^3^ Department of Surgery, Endocrine Surgery Unit, IRCCS San Raffaele Scientific Institute, Milan, Italy; ^4^ Medical Genetics, IRCCS San Raffaele Scientific Institute, Milan, Italy; ^5^ Pathology Unit, IRCCS San Raffaele Scientific Institute, Milan, Italy

**Keywords:** case report, PTEN hamartoma tumor syndrome, congenital hypothyroidism, TPO, goiter

## Abstract

We report the case of a paediatric female patient affected by Bannayan-Riley-Ruvalcaba syndrome (BRRS) and congenital hypothyroidism (CH) with homozygous mutation of the TPO gene. She underwent total thyroidectomy at the age of seven years because of the development of a multinodular goiter. BRRS patients present an increased risk of benign and malignant thyroid disease since childhood because of inactivating mutation of PTEN, an onco-suppressor gene. Instead, homozygous mutations in the TPO gene can be associated with severe forms of hypothyroidism with goiter; previous studies have described cases of follicular and papillary thyroid cancer in CH patients with TPO mutation despite a perfectly controlled thyroid function with Levothyroxine therapy. To our knowledge, this is the first case that describes the possible synergic role of coexisting mutation of both TPO and PTEN in the development of multinodular goiter underlining the importance of a tailored surveillance program in these patients, especially during childhood.

## 1 Introduction

Bannayan-Riley-Ruvalcaba syndrome (BRRS) is caused by germline inactivating mutations of phosphatase and tensin homolog (PTEN) gene inherited with an autosomal dominant mechanism. The PTEN gene, located on chromosome 10q23, is a tumor suppressor gene with a fundamental role in cell growth, migration, and apoptosis via the PI3K/AKT/mTOR pathway (1). An excessive activation of this pathway leads to overgrowth and an increased risk for tumor development as described in PTEN hamartoma tumor syndrome (PHTS), which encompass not only BRRS but a spectrum of syndromes including Cowden syndrome, Lhermitte-Duclos syndrome, Proteus-like syndrome and autism spectrum diseases with macrocephaly (2). BRRS is considered the PHTS form of childhood (3). Macrocephaly (head circumference > 97° ple) is the hallmark of this syndrome (4), variably associated with developmental delay, lipomas, hemangiomas, intestinal hamartomatous polyps, penile freckling and other highly variable phenotypic features as downward slanting palpebral fissures, frontal bossing, macrosomia, café au lait spots, hypotonia, joint hyperextensibility, hypoglycemia and seizures (3, 5). All patients are at high risk of tumor development especially affecting endometrium, kidneys, skin and thyroid, therefore cancer surveillance programs are recommended and needed also for paediatric population (6, 7). Thyroid diseases are common in these children: two thirds of all BRRS patients present with autoimmune thyroiditis, nodular goiter and benign or malignant thyroid tumors, either follicular or papillary thyroid carcinoma (1). In this paper, we describe the case of a girl affected by BRRS presenting with multiple thyroid nodules, previously diagnosed with severe congenital hypothyroidism (CH) due to TPO gene mutation: as far as we know this is the first description of BRRS associated with congenital hypothyroidism.

## 2 Case presentation

**Figure 1:**
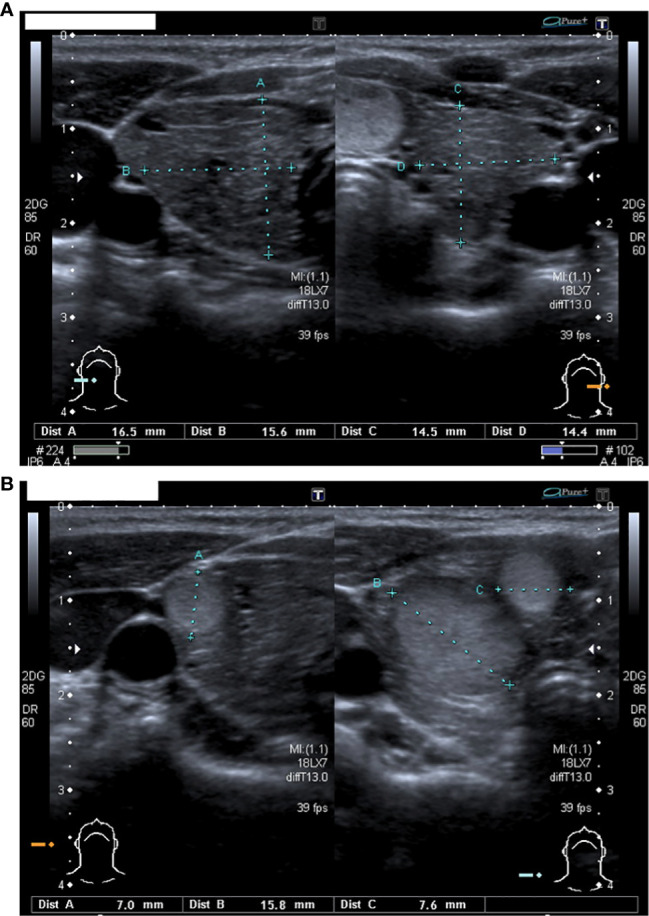
**(A)** The thyroid ultrasound performed at 6 years of age: an enlarged and non-homogeneous echotexture gland with increased antero-posterior and transversal diameters (16,5*15,6 mm for right lobe and 14,5*14,4 mm for left lobe). **(B)** Nodules in the right lobe.

Our patient was born at 31 + 4 gestational weeks because of premature rupture of membranes. At birth her auxological parameters were adequate for gestational age (weight 0,4 SDS, length 1,5 SDS and head circumference 0,6 SDS). In the first days of life she suffered from respiratory distress, neonatal jaundice and patent ductus arteriosus pharmacologically closed. Due to prematurity, cerebral ultrasound was performed, resulting within limits. At neonatal screening, she was detected with a high blood TSH value (152 microU/mL). A severe form of congenital hypothyroidism was subsequently diagnosed (TSH 1016 μIU/mL, fT4 <0,4 ng/dL) and at ten days of life Levothyroxine (LT4) was started at a dosage of 10 mcg/kg/day. The thyroid ultrasound revealed the presence of a hyperplastic gland with inhomogeneous echotexture in the absence of thyroid antibodies. Genetic analysis was performed, revealing the presence of a homozygous mutation in the TPO gene (ins.GGCC395, exon 8) inherited from both parents. The father presented with normal thyroid function; the mother suffered from non-autoimmune hypothyroidism with right lobe hypoplasia diagnosed at the age of sixteen. At six months of age, an increasing head circumference (>97° percentile) and facial abnormalities such as triangular face, frontal bossing and hyperthelorism were observed. Karyotype was normal (46 XX) while the array-CGH showed the presence of a non-pathological variation in the copy number without chromosomal imbalance (arr(1-22,X)x2). At seven months, a brain MRI was performed showing a minimal amplification of the subarachnoid space and a slight para physiological reduction of the myelinisation signal. During the follow-up, a mild delay in neuromotor development (first steps at 18 months, first words at 24 months) was observed. Since the second year of life, multiple lipomas were diagnosed and subsequently surgically removed. At the age of six, she appeared in good clinical conditions, with regular growth both in height (25° ple) and weight (25° ple) but with a persisting important macrocrania. She also presented with an important thyromegaly, despite well controlled thyroid function values since the very first months of age. Therefore, a neck ultrasound was performed showing the presence of an enlarged thyroid (antero-posterior and transversal diameters: 16,5*15,6 mm right, 14,5*14,4 mm left, **Figure 1A**) with non-homogeneous echotexture and multiple nodules with intrinsic and peripheral vascularisation: five in the right lobe (diameter: 7-16 mm, **Figure 1B**) and four in the left lobe (diameter: 6-15mm). Six months later the nodules were increasing both in number and size: six-seven in the right lobe (diameter: 4-19 mm) and six in the left lobe (diameter: 4-17 mm). Moreover, brain MRI was repeated revealing the presence of a bone alteration in the orbital roof (1,8-2 cm) suspicious for haemangioma. Because of all the clinical peculiarities presented (macrocrania, neuromotor delay, facial abnormalities, lipomas, thyroid nodules, doubt of hemangioma), after genetic counselling, the sequencing analysis of PTEN gene was performed showing the presence of a heterozygous mutation (c.635-1G>C) coding for a truncated protein causing Bannayan-Riley-Ruvalcaba syndrome (BRRS). In consideration of the increased risk of developing thyroid cancer, the patient underwent total thyroidectomy at the age of seven; the histological exam showed 21 adenomatous nodules (0.1 to 1.7 centimetres) with microfollicular and trabecular architecture and poor in colloid. In 8/8 nodules analysed, the immunohistochemical analysis revealed a loss of nuclear expression of PTEN. Later in the follow-up, she developed an arteriovenous malformation at her right ankle that was surgically removed and genetically analysed confirming the presence of the PTEN gene alteration. Finally, at the latest follow-up (12 years), a breast nodule was observed and confirmed at the ultrasound, as well as duodenal and colic polyps at the endoscopic exam performed because of the presence of faecal occult blood.

## 3 Discussion

PTEN hamartoma tumor syndrome (PHTS) patients present an increased risk of developing benign and malignant thyroid disease. Nodules, goiter and autoimmune thyroiditis have been described in up to 75% of all patients affected by PHTS, including children with BRRS (1, 2). Previous studies have demonstrated that patients with PTEN gene mutations present a cumulative lifetime risk of developing thyroid carcinoma ranging from 21 to 38%, in particular the risk of paediatric differentiated thyroid carcinoma (DTC) is estimated between 4 and 12% with an incidence of 5% from the age of ten (8–11). Therefore, considering the early involvement of the thyroid gland and the possible development of thyroid cancer, clinical and ultrasound surveillance should be performed early in childhood. However, a clear consensus regarding both the time of initiation and the timepoints of thyroid cancer surveillance in paediatric patients has not been established yet. Although the youngest patient described in literature with PHTS and thyroid carcinoma is a four years old boy (10), most authors have suggested that thyroid disease surveillance should begin at age of seven or ten (10, 11). As described by Smith et al. in their retrospective study on 64 children with PHTS, 44% of patients undergoing thyroid ultrasound present with a clinically significant thyroid nodule at the mean age of 13.3 years with a later presentation in males than females according to gender pubertal period. In this cohort, nodules were rare before the age of seven years (11). On the contrary, in a recent study conducted on a small group of 12 children with PTHS, 41.7% of subjects with nodular thyroid disease were less than seven years old (3). Nonetheless, confirming the prevalence of benign nodular disease in children with PHTS, no patient developed DCT during an average follow-up period of five years, thus not suggesting the need for anticipating the starting age of thyroid cancer surveillance (3). According to the recent comprehensive review of the German paediatric guidelines by Plamper et al., patients with PHTS should undergo a comprehensive physical examination and thyroid ultrasound right after the diagnosis; the following thyroid ultrasound should then be repeated annually or more frequently in case of any suspicious result. Furthermore, children under the age of seven without proof of thyroid nodules are allowed to repeat an ultrasound after 2-3 years (1, 12). Another recent prospective study investigated the development and progression of thyroid nodules and DTC in patients with PHTS both in the presence and absence of thyroid disease at initial ultrasound, with the aim of providing stronger evidence to refine current surveillance recommendations and stratify surveillance intervals based on the initial ultrasound result (13). According to these authors, in contrast with current recommendations, patients without thyroid nodules at the first ultrasound should repeat the exam after 3-5 years; patients with nodules should instead repeat the ultrasound depending on the presence of any suspicious pattern (13). Despite the optimal thyroid function values with LT4 therapy, our patient presented with an important thyromegaly and from the age of six multiple thyroid nodules were described. Homozygous mutations in TPO gene, inherited with an autosomal recessive pattern, are associated with severe forms of hypothyroidism with goiter (14). The combination of CH and DTC is a rare condition with no established causal relationship. As recently suggested by Penna et al., the long exposure to elevated serum TSH levels, a congenital goiter as well as the presence of stimulating factors or the absence of tumor suppressor genes, the type of mutation and the iodine intake could all be factors involved in the development of DTC in patients with CH due to dyshormonogenesis (15, 16). Previous studies have described cases of follicular and papillary thyroid carcinoma in CH patients with TPO mutation and a perfectly controlled thyroid function (17, 18) suggesting that genetic and environmental factors other than TSH level might be involved in the development of thyroid cancer in dyshormonogenic multinodular goiter (MNG) and perhaps play a synergic role in the development of DTC. Tobias et al. analysing the long-term outcome of 33 CH patients with TPO mutations showed that 61% of them developed MNG over time at an average age of 8,6 years (19) without an association with higher TSH levels. In this case series, thyroidectomy was performed in eight patients (24%) leading to the diagnosis of a minimally invasive follicular carcinoma and seven cases of follicular hyperplasia or adenoma (19). Thus, the high rate of MNG development and the risk for thyroid carcinoma indicates the need for regular ultrasound and a long-term follow up in these patients (19). Although most patients affected by PTHS develop benign nodular goiter (3, 20, 21), our patient underwent total thyroidectomy at the age of seven without previously performing a cytological investigation by fine-needle aspiration. According to recent studies, the direct surgical approach could be justified because fine needle biopsy may not be sufficient to exclude malignancy in patients with more than one suspicious lesion (1). Nevertheless, paediatric patients, even more PTHS children with neuromotor delay, are often uncooperative (22) and most cases need general anaesthesia for FNA (1). Furthermore, the cytological analysis does not allow to distinguish a benign follicular adenoma from follicular carcinoma (23). Therefore, prophylactic thyroidectomy should be considered in selected patients with multiple nodules (24). However in the decision-making process, the consequences of total thyroidectomy should be always carefully analysed, such as the need of a lifelong daily replacement therapy and the risk of a surgical procedure in patients with other comorbidities. When indicated, thyroidectomy should be performed by an expert surgeon to minimize the risk of complications.

In conclusion, this is an original case where TPO and PTEN mutation could have played a synergic role in the development of multinodular goiter. Both TPO and PTEN patients need a strict ultrasound follow-up because of the risk of benign and malignant thyroid carcinoma. Further studies are needed to better understand the natural history of thyroid involvement in patients with TPO and/or PTEN mutations in order to provide stronger evidence to refine paediatric surveillance recommendations and develop a tailored diagnostic and therapeutic approach for these patients.

## Data availability statement

The original contributions presented in the study are included in the article/supplementary material, further inquiries can be directed to the corresponding author.

## Ethics statement

Written informed consent was obtained from the minor(s)’ legal guardian/next of kin for the publication of any potentially identifiable images or data included in this article.

## Author contributions

All authors listed have made a substantial, direct, and intellectual contribution to the work and approved it for publication.
